# Clinical implications of peripheral blood biomarkers in patients with advanced breast cancer treated with trastuzumab emtansine and trastuzumab deruxtecan

**DOI:** 10.1007/s10147-025-02768-4

**Published:** 2025-04-29

**Authors:** Yusa Togashi, Masayuki Nagahashi, Aoi Oshiro, Gen Sugimoto, Ayumu Mitsuyoshi, Haruka Kanaoka, Akira Hattori, Junko Tsuchida, Tomoko Higuchi, Arisa Nishimukai, Yasuo Miyoshi

**Affiliations:** Department of Surgery, Division of Breast and Endocrine Surgery, School of Medicine, Hyogo Medical University, 1-1 Mukogawa-cho, Nishinomiya, Hyogo 663-8501 Japan

**Keywords:** Breast neoplasm, Peripheral blood biomarker, Neutrophil-to-lymphocyte ratio, Human epidermal growth factor receptor 2, Antibody–drug conjugate

## Abstract

**Background:**

Development of antibody–drug conjugates, including trastuzumab emtansine (T-DM1) and trastuzumab deruxtecan (T-DXd), has improved the outcomes of patients with HER2-positive breast cancer. We compared the association between peripheral blood biomarkers and outcomes in patients with breast cancer treated with T-DM1 and T-DXd.

**Methods:**

Eighty-five women treated with T-DM1 (*n* = 40) or T-DXd (*n* = 45) for advanced disease were evaluated. Overall survival (OS) and OS after the end of treatment (EOT) were compared based on changes in absolute lymphocyte count (ALC) and neutrophil-to-lymphocyte ratio (NLR) between baseline and EOT.

**Results:**

In the T-DM1 group, patients with a low NLR at EOT had significantly longer OS after EOT than those with a high NLR (*p* = 0.007), and patients with a high ALC at EOT had longer OS after EOT (*p* = 0.071). In the T-DXd group, the ALC and NLR were not associated with OS. The exploratory subgroup analysis suggested that patients with high ALC at EOT had better OS after EOT (*p* = 0.038) in the T-DXd (HER2-low) group (*n* = 19), whereas ALC and NLR were not associated with the outcome in the T-DXd (HER2-positive) group (*n* = 26). Multivariable analysis revealed that the NLR at EOT was an independent prognostic factor for OS after EOT, after adjusting for clinicopathological factors, in the T-DM1 group (*p* = 0.019).

**Conclusion:**

Immune status may influence treatment outcomes in the T-DM1 and T-DXd (HER2-low) groups. Conversely, in the T-DXd (HER2-positive) group, the treatment outcome was independent of immune status.

**Supplementary Information:**

The online version contains supplementary material available at 10.1007/s10147-025-02768-4.

## Introduction

Approximately 15–20% of breast cancers are classified as human epidermal growth factor receptor 2 (HER2)-positive, which was initially considered among the most aggressive forms of the disease [1, 2]. However, with the development of trastuzumab and several subsequent molecular-targeted drugs against the HER2 protein kinase, the outcomes of patients with HER2-positive breast cancer have significantly improved [3]. In recent years, the introduction of antibody–drug conjugates, such as trastuzumab emtansine (T-DM1) and trastuzumab deruxtecan (T-DXd), has further improved treatment efficacy, thus making HER2-positive breast cancer the subtype with the most notable therapeutic advances [4, 5]. Despite these advancements, HER2-positive breast cancer continues to be life-threatening for many patients. A comprehensive understanding of the biologic mechanisms underlying HER2-positive breast cancer is essential to improve treatment strategies, and understanding its relationship with immunity will be a key factor for improving patient outcomes.

Immunity underlies the mechanism of action of drug therapies against breast cancer. For example, tumor-infiltrating lymphocytes (TILs) are associated with outcomes in patients with HER2-positive breast cancer [6, 7]. This finding suggests that antitumor immunity in the tumor microenvironment contributes to drug efficacy in this subtype. Specifically, in the early setting, in patients with HER2-positive breast cancer undergoing preoperative chemotherapy, a higher TIL score correlates with an increased probability of achieving a pathological complete response [8, 9]. However, in the metastatic setting, metastatic lesion biopsies are challenging, because a histopathological diagnosis is required to examine the tumor immune microenvironment, including TILs. Therefore, there are patients in whom the tumor immune microenvironment cannot be directly evaluated; therefore, alternative diagnostic methods are required.

Peripheral blood biomarkers such as the absolute lymphocyte count (ALC) and neutrophil-to-lymphocyte ratio (NLR) are readily obtained from routine clinical blood draws and are useful indicators of patient immunity [10]. Among the peripheral blood biomarkers, ALC correlates with antitumor immunity, whereas NLR is a prognostic indicator of the interplay between immunologic and inflammatory processes [11]. Peripheral blood biomarkers, including ALC and NLR, can predict treatment and prognosis for various breast cancer therapies [12–20]. For example, in patients with HER2-positive breast cancer, a low NLR at the start of T-DM1 treatment is associated with poor treatment outcomes [21]. However, the relationship between peripheral blood markers and treatment outcomes in patients treated with T-DXd has not yet been fully elucidated. Therefore, we aimed to compare the association between peripheral blood biomarkers at baseline and end of treatment (EOT) and outcomes in patients treated with T-DM1 and T-DXd. We assessed whether peripheral blood biomarkers were associated with treatment outcomes in patients with low-HER2 breast cancer treated with T-DXd and whether the relationship between immunity and treatment outcome differed according to HER2 expression status.

## Patients and methods

### Patient eligibility

Eighty-five women who received treatment with T-DM1 (*n* = 40) or T-DXd (*n* = 45) for advanced or metastatic breast cancer at the Hyogo College of Medicine Hospital between January 2011 and July 2024 were assessed. In the T-DXd group, 26 patients had HER2-positive breast cancer, and 19 had HER2-low-expressing breast cancer. The clinical characteristics of the patients are presented in Supplementary Table 1. The patient cohort comprised participants from a previous study [21]. This study was approved by the Institutional Review Board of the Hyogo College of Medicine (No. 1969) and conducted in accordance with the Declaration of Helsinki. The requirement for written informed consent was waived because this study involved only retrospective clinical data collection without patient risk.

All patients included in this study were histologically diagnosed with breast cancer. Specimens with ≥ 1% nuclear staining were classified as ER-positive. HER2-positivity was defined based on an immunohistochemistry (IHC) score of 3 + or 2 + and positive *HER2* amplification based on in situ hybridization (ISH) analysis. HER2-low expression was defined based on an IHC score of 2 + and negative *HER2* amplification by ISH or an IHC score of 1 + .

### Treatment procedures and outcome evaluations

T-DM1 and T-DXd were intravenously infused at 3.6 mg/kg and 5.4 mg/kg over a 3-week cycle, respectively. The treatment was terminated in patients with disease progression, unacceptable toxicity, or death. If a patient experienced toxic side effects related to any of these agents, the dose was interrupted, reduced, or delayed according to a predefined dose adjustment protocol under physician guidance.

Progression-free survival (PFS) was defined as the time from the initiation of T-DM1 or T-DXd therapy to disease progression or death from any cause. Overall survival (OS) was defined as the time from the start of T-DM1 or T-DXd treatment to death from any cause. The OS after EOT was defined as the time from EOT with T-DM1 or T-DXd treatment to death from any cause.

### Measurements of ALC and NLR

Neutrophil and lymphocyte counts were determined automatically using a Sysmex hematology analyzer XN-9000 (Sysmex Corporation, Kobe, Japan) at the Hyogo College of Medicine Hospital. For each patient, the NLR was calculated by dividing the neutrophil count by the lymphocyte count. Peripheral blood biomarker data at baseline were obtained from blood collected before the first administration of T-DM1 or T-DXd, whereas peripheral blood biomarker data at the EOT were obtained from blood collected nearest to the EOT with T-DM1 or T-DXd. Based on a previous study, the ALC and NLR threshold values were set at 1,000/μL and 3, respectively [22].

### Statistical analysis

Kaplan–Meier curves and log-rank tests were used for each group to assess PFS and OS. A Cox proportional hazards model was used to conduct univariable and multivariable analyses of OS, yielding hazard ratios (HR) and 95% confidence intervals (CI). The threshold for statistical significance was established at *p* < 0.05, using a two-sided t test. All statistical analyses were performed using JMP Pro 17 software (SAS Institute Inc., Cary, NC, USA).

## Results

### Clinical outcomes of patients treated with T-DM1 or T-DXd according to ALC and NLR at baseline

**Fig. 1:**
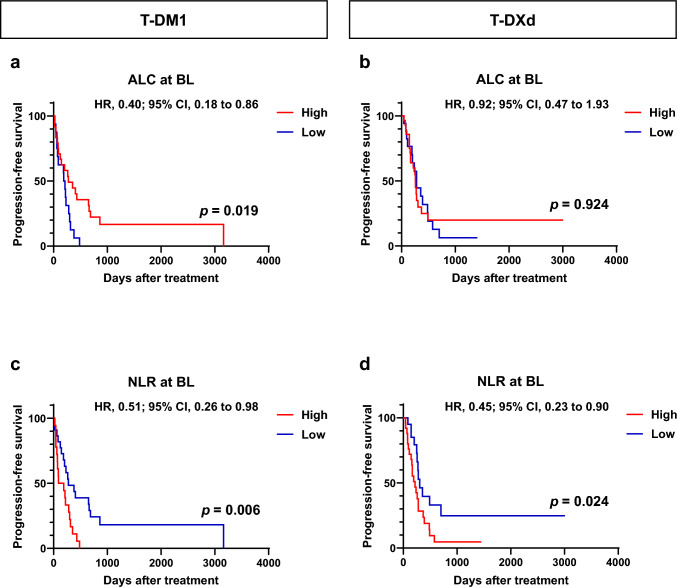
Kaplan–Meier plot of progression-free survival in patients with breast cancer treated with trastuzumab emtansine (T-DM1) or trastuzumab druxtecan (T-DXd) according to absolute lymphocyte count (ALC) and neutrophil-to-lymphocyte ratio (NLR) at baseline (BL). (**a**, **b**) Kaplan–Meier plot of progression-free survival for patients with breast cancer treated with T-DM1 (*n* = 40, **a**) and T-DXd (*n* = 45, **b**) according to ALC at BL. **c**, **d** Kaplan–Meier plot for progression-free survival for patients with breast cancer treated with T-DM1 (*n* = 40, **c**) and T-DXd (*n* = 45, **d**) according to NLR at BL. *HR* hazard ratio; *CI* confidence interval

**Fig. 2:**
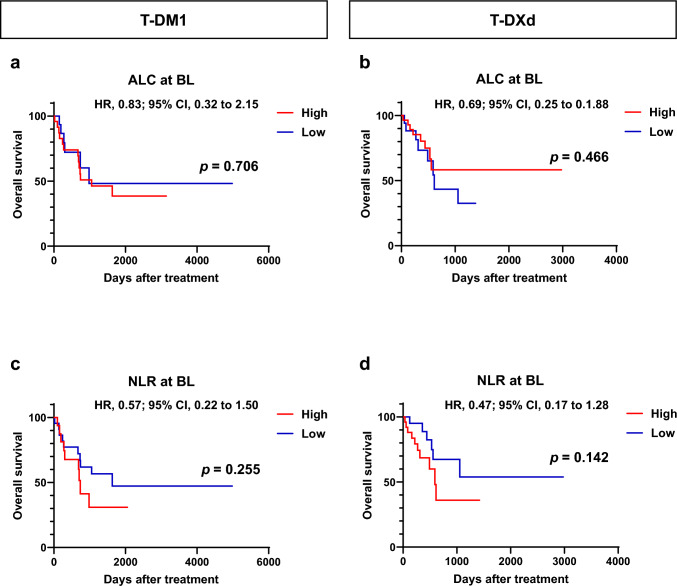
Kaplan–Meier plot for overall survival in patients with breast cancer treated with trastuzumab emtansine (T-DM1) or trastuzumab druxtecan (T-DXd) according to absolute lymphocyte count (ALC) and neutrophil-to-lymphocyte ratio (NLR) at baseline (BL). **a**, **b** Kaplan–Meier plot for the overall survival for patients with breast cancer treated with T-DM1 (*n* = 40, **a**) and T-DXd (n = 45, **b**) according to ALC at BL. **c**, **d** Kaplan–Meier plot for overall survival for patients with breast cancer treated with T-DM1 (n = 40, **c**) and T-DXd (n = 45, **d**) according to NLR at BL. *HR* hazard ratio; *CI* confidence interval

We evaluated the association between the clinical outcomes in 85 patients treated with T-DM1 or T-DXd and the peripheral blood biomarkers ALC and NLR at baseline (Fig. 1 and 2). The median PFS for all patients was 258 d (95% CI, 203–298 d), whereas the median OS was 1054 d (95% CI, 688 d; not determined). In the T-DM1 group at baseline, the high ALC and low-NLR groups had significantly better PFS than the low ALC and NLR groups, respectively (*p* = 0.019 and *p* = 0.006, Fig. 1a, c). In the T-DXd group at baseline, the low-NLR group had significantly better PFS than the high-NLR group (*p* = 0.024; Fig. 1d), whereas ALC was not associated with PFS (Fig. 1b). In both the T-DM1 and T-DXd groups, ALC and NLR were not associated with OS (Fig. 2a–d).

### OS after EOT in patients treated with T-DM1 or T-DXd according to ALC and NLR at EOT

**Fig. 3:**
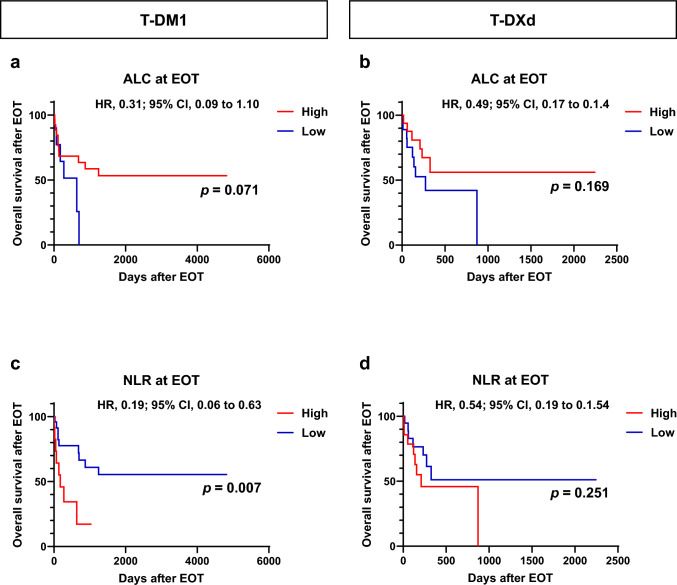
Kaplan–Meier plot for overall survival after end of treatment (EOT) in patients with breast cancer treated with trastuzumab emtansine (T-DM1) or trastuzumab druxtecan (T-DXd) according to absolute lymphocyte count (ALC) and neutrophil-to-lymphocyte ratio (NLR) at EOT. **a**, **b** Kaplan–Meier plot for overall survival after EOT for patients with breast cancer treated with T-DM1 (*n* = 37, **a**) and T-DXd (*n* = 44, **b**) according to ALC at EOT. **c**, **d** Kaplan–Meier plot for overall survival after EOT for patients with breast cancer treated with T-DM1 (n = 37, **c**) and T-DXd (*n* = 44, **d**) according to NLR at BL. *HR* hazard ratio; *CI* confidence interval

We next investigated the relationship between ALC and NLR at EOT and OS after EOT (Fig. 3). The treatment details after EOT for each group are shown in Supplementary Tables 2–4. Notably, in the T-DM1 group, patients with a low NLR at EOT had a significantly longer OS than those with a high NLR at EOT (*p* = 0.007, Fig. 3c). Moreover, patients with a high ALC at EOT had a longer OS after EOT than those with a low ALC at EOT (*p* = 0.071, Fig. 3a). In the T-DXd group, the ALC and NLR were not associated with OS (Fig. 3b, d).

### Univariable and multivariable analyses for OS after EOT

As the NLR at EOT was significantly associated with OS after EOT in the T-DM1 group, we examined whether the NLR at EOT was an independent prognostic factor in this group (Table 1). The univariable analysis revealed that NLR at EOT (low vs. high) was a significant prognostic factor for OS after EOT (*p* = 0.011; Table 1). The NLR at EOT (low vs. high) was also a significant and independent prognostic factor for OS after EOT, after adjusting for other clinicopathological factors (*p* = 0.019, Table 1).

**Table 1:** Univariable and multivariable analyses of overall survival after EOT in patients treated with T-DM1

	*n*	Univariable analysis		Multivariable analysis	
		HR (95% CI)	*p* value	HR (95% CI)	*p* value
Menopausal status					
Premenopausal	8	1.000		1.000	
Postmenopausal	32	2.265 (0.522–9.817)	0.275	3.898 (0.324–46.923)	0.284
Primary advanced or recurrence					
Primary advanced	18	1.000		1.000	
Recurrence	22	1.716 (0.673–4.374)	0.258	3.033 (0.844–10.897)	0.089
Metastatic sites at EOT					
Nonvisceral	15	1.000		1.000	
Visceral	25	1.553 (0.588–4.103)	0.375	1.293 (0.407–4.111)	0.663
Brain metastasis					
None	29	1.000		1.000	
Present	11	1.433 (0.538–3.817)	0.472	2.050 (0.403–10.423)	0.387
Treatment line					
1st or 2nd	20	1.000		1.000	
≥ 3rd	20	2.211 (0.839–5.827)	0.109	1.727 (0.516–5.778)	0.375
Disease progression at EOT					
None	12	1.000		1.000	
Present	25	0.490 (0.196–1.221)	0.126	1.162 (0.347–3.889)	0.808
New lesion at EOT					
None	27	1.000		1.000	
Present	10	0.280 (0.065–1.214)	0.089	0.160 (0.025–1.035)	0.054
ALC at EOT					
Low	10	1.000		1.000	
High	27	0.390 (0.135–1.123)	0.081	2.021 (0.358–11.404)	0.425
NLR at EOT					
Low	25	1.000		1.000	
High	12	3.794 (1.362–10.574)	0.011	253 (1.357–28.807)	0.019

*EOT* end of treatment; *T-DM1* trastuzumab emtansine; *CI* confidence interval; *ALC* absolute lymphocyte count; *NLR* neutrophil-to-lymphocyte ratio

### Clinical outcomes in patients with HER2-positive and HER2-low-expressing breast cancer in the T-DXd group according to ALC and NLR at baseline and EOT

**Fig. 4:**
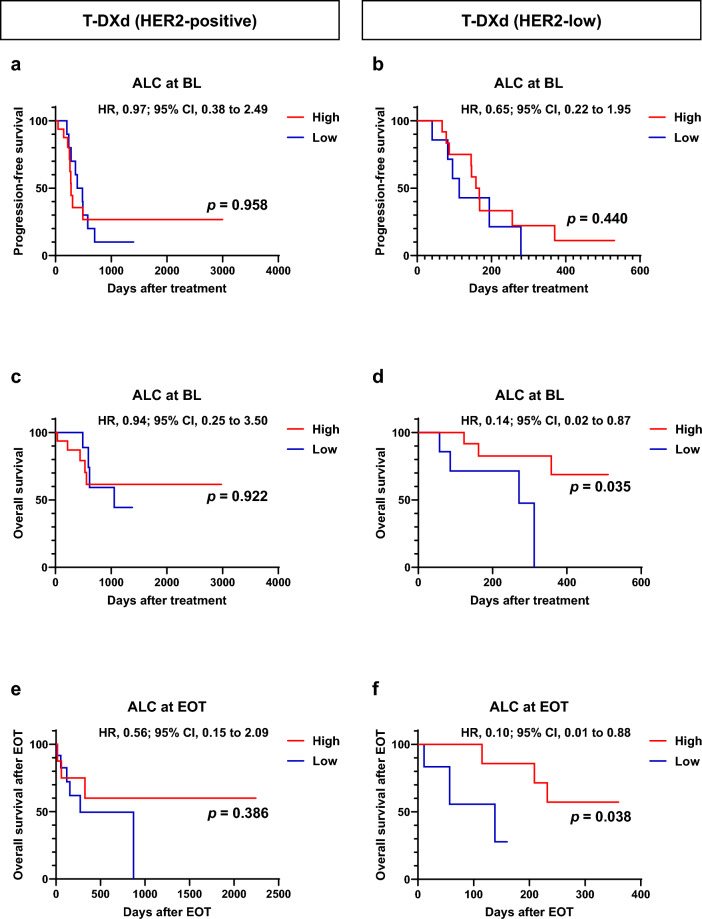
Kaplan–Meier plot of progression-free and overall survival in patients with HER2-positive and HER2-low breast cancer treated with trastuzumab druxtecan (T-DXd) according to absolute lymphocyte count (ALC) at baseline (BL) and end of treatment (EOT). **a**, **b** Kaplan–Meier plot for progression-free survival for patients with HER2-positive (*n* = 26, a) and HER2-low (*n* = 19, **b**) breast cancer treated with T-DXd according to ALC at BL. **c**, **d** Kaplan–Meier plot for overall survival for patients with HER2-positive (*n* = 26, **c**) and HER2-low (*n* = 19, **d**) breast cancer treated with T-DXd according to ALC at BL. **e**, **f** Kaplan–Meier plot for overall survival after EOT for patients with HER2-positive (*n* = 26, **e**) and HER2-low (*n* = 19, **f**) breast cancer treated with T-DXd according to ALC at EOT. *HR* hazard ratio; *CI* confidence interval

**Fig. 5:**
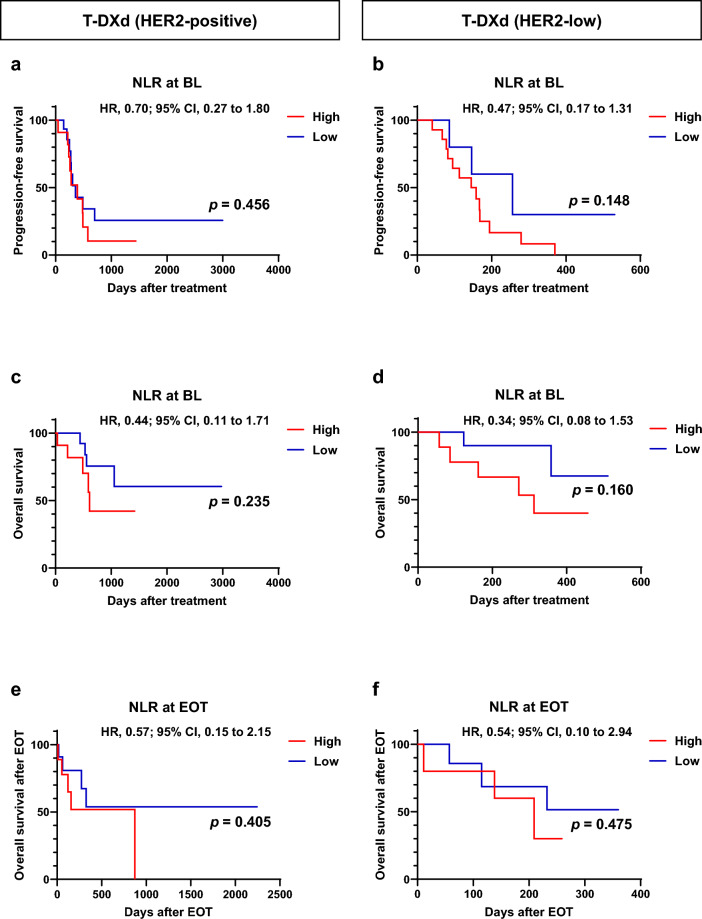
Kaplan–Meier plot of progression-free and overall survival in patients with HER2-positive and HER2-low breast cancer treated with trastuzumab druxtecan (T-DXd) according to neutrophil-to-lymphocyte ratio (NLR) at baseline (BL) and end of treatment (EOT). (**a**, **b**) Kaplan–Meier plot for progression-free survival for patients with HER2-positive (*n* = 26, **a**) and HER2-low (*n* = 19, **b**) breast cancer treated with T-DXd according to NLR at BL. (**c**, **d**) Kaplan–Meier plot for overall survival for patients with HER2-positive (*n* = 26, **c**) and HER2-low (*n* = 19, **d**) breast cancer treated with T-DXd according to NLR at BL. **e**, **f** Kaplan–Meier plot for overall survival after EOT for patients with HER2-positive (*n* = 26, **e**) and HER2-low (*n* = 19, **f**) breast cancer treated with T-DXd according to NLR at EOT. *HR* hazard ratio; *CI* confidence interval

Next, we explored the relationship between ALC, NLR, and clinical outcomes in patients with HER2-positive and HER2-low-expressing breast cancer separately in the T-DXd group (Figs. 4 and 5). In the HER2-positive group, neither ALC, NLR at baseline, nor EOT were associated with clinical outcomes after T-DXd treatment (Fig. 4a, c, e, 5a, c, e). In contrast, in the HER2-low group, the high ALC group at baseline had significantly better OS than the low ALC group (*p* = 0.035; Fig. 4d), and the high ALC group at EOT had significantly better OS after EOT than the low ALC group (*p* = 0.038; Fig. 4e). Furthermore, the NLR at baseline and EOT were not associated with clinical outcomes in patients with HER2-low breast cancer treated with T-DXd (Fig. 5b, d, f).

## Discussion

We have previously reported that patients with a lower NLR during the initiation of T-DM1 therapy had significantly better PFS, which suggests that the immune environment may contribute to treatment efficacy [21]. However, the relationship between peripheral blood biomarkers and T-DXd efficacy has not been fully elucidated. The results of this study revealed a significant association between peripheral blood biomarkers, both at baseline and at EOT, and treatment outcomes, which suggests that the immune environment affects the treatment outcomes in the T-DM1 group. Our results also suggest that the treatment effect of T-DXd in patients with HER2-positive breast cancer can be achieved regardless of the immune environment. The immune status demonstrated the prognostic significance of T-DXd treatment in patients with low-HER2 expression. These findings altogether suggest that the effect of immunity on the efficacy of T-DXd therapy depends on HER2 expression.

In the present study, following T-DM1 treatment, ALC and NLR at baseline significantly associated with PFS, and NLR at EOT significantly associated with prognosis after EOT. In HER2-positive breast cancer, antitumor immune responses are associated with therapeutic efficacy [23]. We previously reported that patients with breast cancer treated with CDK4/6 inhibitors who had a favorable immune environment, as indicated by peripheral blood biomarkers at the start of treatment, had better outcomes [20]. Patients who maintained a favorable immune environment throughout treatment also had better outcomes in subsequent treatments [20]. Patients with good immune status throughout treatment showed better subsequent prognosis in the T-DM1 treatment group, as in the CDK4/6 inhibitor treatment group, thus suggesting that the immune environment affects treatment efficacy and prognosis.

Among the patients treated with T-DXd, the low-NLR group had significantly better PFS than the high-NLR group at baseline. Our study also showed that in patients treated with T-DM1, the low-NLR group had significantly better PFS than the high-NLR group, which suggests that NLR is a predictor of prognosis rather than a specific predictor of treatment response for T-DXd. Indeed, NLR has often been reported as a prognostic factor in previous reports, which is consistent with the present results. Although no difference in OS was observed by NLR, the difference in PFS may be generated by the prognostic role of NLR in T-DXd-treated patients.

The exploratory subgroup analysis suggested that neither peripheral blood biomarkers at baseline nor EOT were associated with treatment outcomes in patients with HER2-positive breast cancer treated with T-DXd. While the immune milieu has been suggested to influence T-DM1 therapy as mentioned above, the correlation of TILs with pathologic complete response in preoperative chemotherapy with trastuzumab and pertuzumab in patients with HER2-positive breast cancer suggests that antitumor immunity is also important for treatment outcomes [8, 9]. However, there is a paucity of data regarding the relationship between the therapeutic effects of T-DXd and the immune milieu, including peripheral blood biomarkers and TILs. Considering that T-DXd has a strong antitumor effect on HER2-positive breast cancer cells [24], it may be effective regardless of the patient’s immunity.

An exploratory analysis also suggested that ALC was associated with OS in patients with HER2-low breast cancer treated with T-DXd. This observation may be due to the relatively weak effect of T-DXd in HER2-low-expressing breast cancer, unlike in HER2-positive breast cancer, where there may be an immune response. Few studies have reported the therapeutic effect of T-DXd on HER2-low-expressing breast cancer and the immune environment. Investigating whether the mechanism of action of T-DXd depends on HER2 expression levels merits attention.

This study had some limitations that warrant further consideration. First, this was a retrospective study involving a small number of patients at a single institution. In the T-DXd group, we performed a subgroup analysis to examine the clinical impact of ALC and NLR in the HER2-positive and HER2-low groups; however, this subgroup analysis was performed on an exploratory basis owing to multiple comparisons. Furthermore, the subgroup analysis was performed with a very small number of patients. Because this was a small study, the findings need to be further validated across a larger and more diverse study populations in future. Second, almost half of the patients were treated with T-DM1 before T-DXd in the HER2-positive group, and historical differences may have resulted in different patient backgrounds and treatment options.

Nonetheless, this study showed a consistent trend: immune status at the beginning and EOT were associated with patient outcomes in T-DM1 treatment, whereas immune status at the beginning and EOT were not associated with patient outcomes in T-DXd treatment for HER2-positive breast cancer, thus clearly distinguishing both treatments. Moreover, the data suggested an immune influence of T-DXd treatment in HER2 low-expressing breast cancer, which suggests different mechanisms of action of T-DXd treatment that depend on HER2 expression in breast cancer.

In conclusion, our findings indicate that the immune status at baseline and EOT affected treatment outcomes in patients treated with T-DM1, whereas the treatment efficacy of T-DXd in patients with HER2-positive breast cancer can be achieved regardless of immune status. Immune status was associated with treatment outcomes in patients with HER2-low breast cancer treated with T-DXd, which suggests that the relationship between immunity and T-DXd treatment outcomes differ according to HER2 expression. Further studies are warranted to investigate the contribution of immunity to the mechanism of action of these drugs.

## Supplementary Information

Below is the link to the electronic supplementary material.

Supplementary file1 (PDF 316 KB)

## Data Availability

The datasets used in this study are available from the corresponding author upon request.
