# Supplementary material for: Clinical implications of peripheral blood biomarkers in patients with advanced breast cancer treated with trastuzumab emtansine and trastuzumab deruxtecan
Source: Int J Clin Oncol. 2025 Apr 29;30(7):1331–40. doi: 10.1007/s10147-025-02768-4 (PMC12187903; doi:10.1007/s10147-025-02768-4)
Supplement: Supplementary file 1 — Supplementary file1 (PDF 316 KB) [file 10147_2025_2768_MOESM1_ESM.pdf]

Supplementary Table 1: Clinical characteristics of patients treated with T-DM1 or T-DXd

	T-DM1<br>*n* = 40	T-DXd (HER2-positive)<br>*n* = 26	T-DXd (HER2-low)<br>*n* = 19
Age, median (range)	57 (34-91)	57 (28-86)	57 (36-84)
Menopausal status			
Premenopausal	8 (20.0%)	7 (26.9%)	7 (36.8%)
Postmenopausal	32 (80.0%)	19 (73.1%)	12 (63.2%)
Primary advanced or recurrence			
Primary advanced	18 (45.0%)	6 (23.1%)	10 (52.6%)
Recurrence	22 (55.0%)	20 (76.9%)	9 (47.4%)
Metastatic sites			
Non-visceral	15 (37.5%)	3 (11.5%)	0 (0.0%)
Visceral	25 (62.5%)	23 (88.5%)	19 (100.0%)
Brain metastasis			
None	29 (72.5%)	15 (57.7%)	14 (73.7%)
Present	11 (27.5%)	11 (42.3%)	5 (26.3%)
Lung metastasis			
None	27 (67.5%)	15 (57.7%)	8 (42.1%)
Present	13 (32.5%)	11 (42.3%)	11 (57.9%)
Liver metastasis			
None	23 (57.5%)	15 (57.7%)	6 (31.6%)
Present	17 (42.5%)	11 (42.3%)	13 (68.4%)
Bone metastasis			
None	16 (40.0%)	11 (42.3%)	7 (36.8%)
Present	24 (60.0%)	15 (57.7%)	12 (63.2%)
Treatment line			
1st	6 (15.0%)	2 (7.7%)	0 (0.0%)
2nd	14 (35.0%)	7 (26.9%)	3 (15.8%)
≥3rd	20 (50.0%)	17 (65.4%)	16 (84.2%)
T-DXd before T-DM1			
None	40 (100.0%)	NA	NA
Present	0 (0.0%)	NA	NA
T-DM1 before T-DXd			
None	NA	15 (57.7%)	19 (100.0%)
Present	NA	11 (42.3%)	0 (0.0%)

T-DM1, trastuzumab emtansine; T-DXd, trastuzumab deruxtecan; NA, not associated

Supplementary Table 2: Treatment after T-DM1 in breast cancer patients (*n* = 40)

No. of patients	1st line after T-DM1	2nd line after T-DM1	3rd line after T-DM1	4th line after T-DM1
22	No treatment (BSC)			
6	T-DXd	No treatment (BSC)		
3	PTX+Bev	No treatment (BSC)		
2	ERI	No treatment (BSC)		
1	Nab-PTX	No treatment (BSC)		
1	VNR+T-mab+P-mab	No treatment (BSC)		
1	LET	No treatment (BSC)		
3	T-DXd	ERI	No treatment (BSC)	
1	ERI	PTX+Bev	No treatment (BSC)	
1	LET	Lapa+Cape	ERI	No treatment (BSC)

T-DM1, trastuzumab emtansine; BSC, best supportive care; T-DXd, trastuzumab deruxtecan; PTX, paclitaxel; Bev, bevacizumab; ERI, eribulin; Nab-PTX, nab-paclitaxel; VNR, vinorelbine; T-mab, trastuzumab; P-mab, pertuzumab; LET, letrozole; Lapa, lapatinib; Cape, capecitabine

Supplementary Table 3: Treatment after T-DXd in HER2-positive breast cancer patients (*n* = 26)

No. of patients	1st line after T-DXd	2nd line after T-DXd	3rd line after T-DXd	4th line after T-DXd
17	No treatment (BSC)			
4	ERI	No treatment (BSC)		
2	PTX+Bev	No treatment (BSC)		
1	ERI	EC	No treatment (BSC)	
1	GEM+CBDCA+Pem	ERI	No treatment (BSC)	
1	ERI	FEC	Cape	No treatment (BSC)

T-DXd, trastuzumab deruxtecan; BSC, best supportive care; ERI, eribulin; PTX, paclitaxel; Bev, bevacizumab; EC, epirubicin and cyclophosphamide; GEM, gemcitabine; CBDCA, carboplatin; Pem, pembrolizumab; FEC, fluorouracil, epirubicin and cyclophosphamide; Cape, capecitabine

Supplementary Table 4: Treatment after T-DXd in HER2-low breast cancer patients (*n* = 19)

No. of patients	1st line after T-DXd	2nd line after T-DXd	3rd line after T-DXd
12	No treatment (BSC)		
2	EC	No treatment (BSC)	
1	CMF	No treatment (BSC)	
1	PTX+Bev	No treatment (BSC)	
1	PTX+Bev	CMF	No treatment (BSC)
1	EC	PTX+Bev	No treatment (BSC)
1	Cape	CMF	No treatment (BSC)

T-DXd, trastuzumab deruxtecan; BSC, best supportive care; EC, epirubicin and cyclophosphamide; CMF, cyclophosphamide, methotrexate and fluorouracil; PTX, paclitaxel; Bev, bevacizumab; Cape, capecitabine
